# A RLS-SVM Aided Fusion Methodology for INS during GPS Outages

**DOI:** 10.3390/s17030432

**Published:** 2017-02-24

**Authors:** Yiqing Yao, Xiaosu Xu

**Affiliations:** Key Laboratory of Micro-Inertial Instrument and Advanced Navigation Technology, Ministry of Education, School of Instrument Science and Engineering, Southeast University, Nanjing 210096, China; yucia@sina.com

**Keywords:** INS/GPS integrated navigation system, GPS outage, robust LS-SVM, outlier

## Abstract

In order to maintain a relatively high accuracy of navigation performance during global positioning system (GPS) outages, a novel robust least squares support vector machine (LS-SVM)-aided fusion methodology is explored to provide the pseudo-GPS position information for the inertial navigation system (INS). The relationship between the yaw, specific force, velocity, and the position increment is modeled. Rather than share the same weight in the traditional LS-SVM, the proposed algorithm allocates various weights for different data, which makes the system immune to the outliers. Field test data was collected to evaluate the proposed algorithm. The comparison results indicate that the proposed algorithm can effectively provide position corrections for standalone INS during the 300 s GPS outage, which outperforms the traditional LS-SVM method. Historical information is also involved to better represent the vehicle dynamics.

## 1. Introduction

An inertial navigation system (INS) is a self-contained system with high accuracy over short periods, which has been widely used in military and civil applications, but its performance degrades over time due to the sensors’ errors. Thus, GPS is often integrated with INS by a Kalman filter (KF) to restrain the accumulated positioning error. However, when a vehicle is operating in city canyons, tunnels, and viaducts, where the GPS signal is blocked, the INS/GPS integrated system is forced into the pure INS mode in which the navigation accuracy would deteriorate significantly.

To provide a relatively high-precision navigation solution even during GPS outages, the artificial intelligence (AI)-aided integrated navigation system was proposed and investigated in many studies. The main idea of the solution is that when GPS is available, an AI module is constructed to be trained to find out the relation between the vehicle’s dynamics and some particular information of the integrated system. When GPS is unavailable, the well-trained AI module will be employed to predict the particular information to give corrections to the standalone INS.

Various models were explored in the AI-aided INS/GPS integrated system. According to the different outputs of the AI module, the models can be divided into, and denoted as, the $O_{INS}-\delta P_{INS}$ model, $O_{INS}-X_{k}$ model, and $O_{INS}-\Delta P$ model. Noureldin and his team studied the $O_{INS}-\delta P_{INS}$ model, which relates the measurements from the inertial measurement unit (IMU) to the difference of the outputs of GPS and INS [1,2]. To simplify the algorithm, some other researchers employed the $O_{INS}-X_{k}$ model, which directly maps the function between the INS data and the KF states [3,4]. Recently, the $O_{INS}-\Delta P$ model has arisen to directly explore the relationship between the INS information and the increments of the GPS position [5]. All three models have shown good performance during GPS outages.

The machine learning algorithms for regression in the AI module also vary. The artificial neural network (ANN) algorithms were widely applied in different studies; for example, the radial basis function (RBF) neural network, the adaptive fuzzy neuron-fuzzy inference system, and the wavelet neural network [2,6,7]. However, ANN suffers from the local minimum and over-fitting problems. It is also difficult to choose the number of the layers and the hidden units in an ANN. The LS-SVM regression algorithm, which is based on the structural risk minimisation principle, rather than the minimised empirical error principle implemented in an ANN, shows its advantages in the regression process. The inputs are transformed into a high-dimensional space by LS-SVM to select a hyperplane to present the inner relation between the inputs and the outputs well, which could avoid the problems of over-fitting and local minima [8,9].

Simulations and field tests were operated to evaluate the performance of the proposed models and algorithms in the above studies. The GPS/INS integrated systems were firstly operated in a loosely-coupled mode with good GPS availability and then aided by the AI module during GPS outages. The loosely-coupled period is called the training period, during which the input and output data of the AI module are collected and trained. GPS is assumed with high precision during this period to explore the essential relation between the inputs and outputs. However, although the GPS receiver could indicate the GPS outages, during which the GPS data would not be used for training, there are still outliers among the GPS position data due to the multipath effect or particular motion, which cannot be easily recognized [10]. Involving these outliers in the training set will definitely decrease the effectiveness of the AI module. Thus, new methods should be investigated to reduce the influence of the GPS outliers.

In order to achieve a better navigation performance during the GPS outages, a robust least squares support vector machine (RLS-SVM)-aided INS/GPS integrated system is proposed to overcome the shortcomings of the previous methodologies discussed above, which could depress the effect of the GPS outliers in the training set. Meanwhile, a newly-developed $O_{INS}-\Delta P$ model is adopted, in which the GPS outliers are easier to be determined. The specific force information from the accelerometers, yaw data, and velocity are selected as the inputs of the model, which represents the vehicle dynamics, while the outputs of the model are the increments of the GPS position. When GPS signal is available, the inputs are collected and trained to investigate their relationship with the outputs. Once GPS is unavailable, the information of the INS, which can still be acquired, is utilized to estimate the pseudo-GPS position to correct the navigation results. In addition, past information is also employed to fully represent the vehicle dynamics. To evaluate the effectiveness of the proposed algorithm, field test data is collected and investigated.

The rest of this paper is organized as follows: Section 2 introduces the INS/GPS loosely-coupled integrated navigation system; Section 3 illustrates the proposed RLS-SVM regression methodology; The description of the RLS-SVM aided fusion method is presented in Section 4; Experiments are shown in Section 5 and conclusions are drawn in Section 6.

## 2. INS/GPS Loosely-Coupled Integrated Navigation System

When GPS is available, a 15-state KF is employed to fuse the navigation results of INS and GPS. The state vector X is defined as:

(1) $$X={\left[\begin{array}{ccccccccccccccc} \varphi_{E} & \varphi_{N} & \varphi_{U} & \delta V_{E} & \delta V_{N} & \delta V_{U} & \delta L & \delta \lambda & \delta h & \nabla_{x} & \nabla_{y} & \nabla_{z} & \varepsilon_{x} & \varepsilon_{y} & \varepsilon_{z} \end{array}\right]}^{T},$$

where $\varphi_{E,N,U}$ are the misalignment angles of the calculated platform in the local geographical frame g, $\delta V_{E,N,U}$ are the velocity errors of the three axes of frame g. δL, δλ, and δh denotes the latitude, longitude, and height errors, respectively. $\nabla_{x,y,z}$ and $\varepsilon_{x,y,z}$ represent the accelerometer biases and gyro biases in three directions of the body frame b.

The process model and observation model are:

(2) $$\{\begin{array}{c} \dot{X}=FX+GW\\ Z=HX+V \end{array}$$

where F is the system matrix, G is the system noise matrix, Z is the observation vector, and H is the observation matrix respectively. W and V are the process noise vector and observation noise vector.

The system matrix F can be obtained according to the error equations of the INS:

(3) $${\dot{\varphi }}^{g}=-(\omega_{ig}^{g}\times )\varphi^{g}+\delta \omega_{ig}^{g}-C_{b}^{g}\varepsilon^{b},$$

(4) $$\delta {\dot{V}}^{g}=-((\delta \omega_{ie}^{g}\times )+(\delta \omega_{ig}^{g}\times ))V^{g}-((\omega_{ie}^{g}\times )+(\omega_{ig}^{g}\times ))\delta V^{g}+C_{b}^{g}\nabla^{b}-\left(\varphi^{g}\times \right)f^{b},$$

(5) $$\begin{array}{l} \delta \dot{L}=\frac{\delta V_{N}^{g}}{R}\\ \delta \dot{\lambda }=\frac{\delta V_{E}^{g}}{R}\sec L+\frac{\tan L}{\cos L}\frac{V_{E}^{g}}{R}\delta L\\ \delta h=\delta V_{U} \end{array},$$

among which $\omega_{ie}^{g}$ and $\delta \omega_{ie}^{g}$ are the Earth rate vector and its error, $\omega_{ig}^{g}$ and $\delta \omega_{ig}^{g}$ are the angular rate vector of frame g to the inertial frame i and its error, and $C_{b}^{g}$ is the direction cosine matrix of transformation from frame b to frame g.

Transforming Equation (2) into the discrete time formula:

(6) $$\{\begin{array}{c} X_{k}=\Phi_{k,k-1}X_{k-1}+G_{k}W_{k}\\ Z_{k}=H_{k}X_{k}+V_{k} \end{array},$$

The update and prediction processes are illustrated as follows:

(7) $${\hat{X}}_{k,k-1}=\Phi_{k,k-1}{\hat{X}}_{k-1},$$

(8) $$P_{k,k-1}=\Phi_{k,k-1}P_{k-1}\Phi_{k,k-1}^{T}+G_{k,k-1}Q_{k-1}G_{k,k-1}^{T},$$

(9) $$K_{k}=P_{k,k-1}H_{k}^{T}{\left[H_{k}P_{k,k-1}H_{k}^{T}+R_{k}\right]}^{-1},$$

(10) $${\hat{X}}_{k}={\hat{X}}_{k,k-1}+K_{k}\left[Z_{k}-H_{k}{\hat{X}}_{k,k-1}\right],$$

(11) $$P_{k}=\left[I-K_{k}H_{k}\right]P_{k,k-1},$$

where ${\hat{X}}_{k,k-1}$ is the predicted state estimate and ${\hat{X}}_{k}$ is the updated state estimate. $\Phi_{k,k-1}$ is the state transition matrix of the system from epoch *k* − 1 to *k*. $P_{k,k-1}$ and $P_{k}$ are the predicted estimate covariance and updated estimate covariance respectively. $K_{k}$ is the Kalman matrix. $Q_{k-1}$ and $R_{k}$ are the variance-covariance matrices of the states and observation, which can be calculated as $Q_{k}=E\left[W_{k}W_{k}^{T}\right]$ and $R_{k}=E\left[V_{k}V_{k}^{T}\right]$, respectively [11].

## 3. RLS-SVM Regression Algorithm

LS-SVM is widely employed for nonlinear classification and regression problems. Based on the structural risk minimisation principle, it can minimize the upper bound of the generalization error. Compared to the support vector machine (SVM), the LS-SVM is simpler and easier to apply, whose solution is characterized by a Karush-Kuhn-Tucker (KKT) system [12]. However, rather than select some important support vectors, the LS-SVM utilizes all the training data for regression, which makes the algorithm vulnerable to the outliers in the training set. In real applications, the GPS data may be subject to the outliers even when the satellite signals are in good condition. Thus, a RLS-SVM regression algorithm is provided to eliminate the influence of the outliers in GPS data.

Given the training dataset ${\left\{x_{k},y_{k}\right\}}_{k=1}^{N}$, where *N* denotes the number of the points in the set, $x_{k}$ and $y_{k}$ are the *k*th input vector and output vector, respectively. The objective is to find a nonlinear function estimation using the following representation:

(12) $$f\left(x\right)=\omega^{T}\phi \left(x\right)+b,$$

where ω is the weight vector, *b* is the bias term. ϕ(x) is a nonlinear function, which maps the input vector to a higher dimensional feature space.

To solve the problem, consider the following optimization problem [13]:

(13) $$\underset{\omega ,b,e}{\min}J\left(\omega ,e\right)=\frac{1}{2}\omega^{T}\omega +\frac{1}{2}\gamma \sum_{k=1}^{N}s_{k}e_{k}^{2},$$

such that:

(14) $$\begin{array}{cc} y_{k}=\omega^{T}\phi \left(x_{k}\right)+b+e_{k}, & k=1,\ldots ,N \end{array},$$

where γ is the regularisation parameter with a constant value, $s_{k}$ is the weighting factor of the *k*th point. It can be seen that a smaller $s_{k}$ depresses the importance of $e_{k}$, which makes the *k*th point in the training dataset less significant.

Transform Equation (13) into the corresponding Lagrangian function format as follows:

(15) $$L\left(\omega ,b,e;\alpha \right)=J\left(\omega ,e\right)-\sum_{k=1}^{N}\alpha_{i}\left\{\omega^{T}\phi \left(x_{k}\right)+b+e_{k}-y_{i}\right\},$$

where α and $\alpha_{k}$ are Lagrange multipliers. Calculate the partial derivatives of L(ω,b,e;α), with respect to ω, *b*, $e_{k}$, $\alpha_{k}$, according to KKT and then eliminate ω and $e_{k}$. The solution can be expressed as:

(16) $$\left[\begin{array}{cc} 0 & l_{N}^{T}\\ l_{N} & \Omega +\frac{1}{\gamma }S \end{array}\right]\left[\begin{array}{c} b\\ \alpha \end{array}\right]=\left[\begin{array}{c} 0\\ y \end{array}\right],$$

where $l_{N}=\left[1,\cdots ,1\right]$, $\begin{array}{cc} \Omega_{i,j}=\phi {\left(x_{i}\right)}^{T}\phi \left(x_{j}\right), & i,j=1,\ldots \end{array},N$ and the weight matrix S is given by:

(17) $$S=diag\left\{\frac{1}{s_{1}},\ldots ,\frac{1}{s_{N}}\right\},$$

Applying the Mercer’s condition, the kernel K can be obtained:

(18) $$\begin{array}{cc} K\left(x_{i},x_{j}\right)=\phi {\left(x_{i}\right)}^{T}\phi \left(x_{j}\right), & i,j=1,\ldots \end{array},N,$$

Choose the commonly used radial basis function (RBF) kernels as the kernel function. The output of the RLS-SVM for regression can be represented as:

(19) $$f(x)=\sum_{k=1}^{N}\alpha_{i}K\left(x_{i},x\right)+b=\sum_{k=1}^{N}\alpha_{i}\exp\left(-{\left\|x_{i}-x\right\|}_{2}^{2}/2\sigma^{2}\right)+b,$$

where σ denotes the kernel width.

In the LS-SVM, the weight matrix S is the identity matrix I, which means that all of the training data are of the same importance to build the function for regression. When the outliers exist, the LS-SVM will bring the errors into the system without recognizing them. In order to reduce the importance of the outliers and provide a robust estimation of f(x), $s_{k}$ is constructed according to the residual of each training sample. The whole process contains two steps. Firstly, assuming S=I, calculate α and *b* by Equation (16), and the corresponding residuals of the training samples can be obtained. The slack variable $\xi_{k}$ is introduced for each training point to indicate the deviation of the training sample outside the ε insensitive zone, which is defined as that if the value of $e_{k}$ is within the zone, we consider the *k*th point in the training data set is not likely to be an outlier. Thus, $\xi_{k}$ can be calculated as:

(20) $$\xi_{k}=\left|\left\|y_{k}-f(x_{i})\right\|-\varepsilon \right|,$$

In the theory of statistics, when a point is farther than three times of the standard deviation, it is regarded as an outlier, assuming the dataset is subject to the Gaussian distribution [14]. Thus, when $\xi_{k}$ is larger than three times of the standard deviation of the slack variables, the *k*th point is considered as an outlier and the corresponding $s_{k}$ is set to be $10^{-4}$ to eliminate its roles in the RLS-SVM.

Secondly, after figuring out the obvious outliers in the training dataset, recalculate the α and *b* using the updated S to eliminate the outliers’ effect. Then, apply the robust loss function to modify the weight factor of $e_{k}$ again:

(21) $$s_{k}=\{\begin{array}{ll} 1 & \begin{array}{cc} if & \left|e_{k}/q\right|\leq c_{1} \end{array}\\ \frac{c_{2}-\left|e_{k}/q\right|}{c_{2}-c_{1}} & \begin{array}{cc} if & c_{1}\leq \left|e_{k}/q\right|\leq c_{2} \end{array}\\ 10^{-4} & otherwise \end{array},$$

where $c_{1}$, $c_{2}$ are constants, which are defined as 2.5 and 3, respectively, according to the characteristic of the Gaussian distribution [15]. *q* is a robust estimation of the standard deviation of the LS-SVM error variables $e_{k}$:

(22) $$q=\frac{IQR}{2\times 0.6745},$$

where the interquartile range *IQR* is the difference between the 75th percentile and 25th percentile.

The selection of γ and σ has been fully discussed in the related studies. Various methods have been proposed to solve this problem, such as genetic algorithms and Bayesian learning methods [5,16]. In this work, a simple cross-validation method is employed, which randomly divides the training dataset into two subsets, for training and validation, respectively. Several empirical combination of the tuning parameters are trained with the training subset and validated in the validation subset. The final selection is made where the output of the algorithm reaches the highest accuracy in the validation subset. Practically, when the system (sensors, AI model, etc.) remains the same, the well-selected parameters can be used in many navigation scenarios.

## 4. RLS-SVM Aided INS/GPS Navigation

The main idea of an AI-aided INS/GPS integrated navigation system is to explore the mathematical relationship between the particular navigation information in the INS/GPS integrated system and the vehicle dynamics indicated by the data from IMU and INS, trying to maintain a high navigation accuracy during GPS outages. When the GPS signal is available, the AI module is trained. During the GPS outages, the well-trained AI module is used to predict the demanding information.

Several AI models were proposed to find the relationship, all of which showed good performance in the experiments. Assumptions were made in these studies that the collected data in the training period are of good quality. However, the GPS data cannot always maintain high accuracy. When outliers exist in the GPS data, not only the AI algorithm, but also the model, needs to be carefully selected to avoid the negative effects. The $O_{INS}-\delta P_{INS}$ model is designed to find the relationship between the information of INS and the difference of the outputs of GPS and INS, while the $O_{INS}-X_{k}$ model tries to relate the information of INS to the state vector of KF. The GPS data is mixed with other information in these models, which makes it hard to characterize the GPS information and to separate the outliers. Thus, in order to eliminate the effect of the outliers, the output of the AI module needs to be some particular information that only relates to the GPS data.

In this study, a pseudo-GPS position is predicted by the AI module to avoid estimating a mixture of both INS and GPS information, which can be denoted as the $O_{INS}-\Delta P$ model. The inputs and outputs of the model are selected according to the differential equations of velocity of the INS [17]. The position increment can be calculated as follows:

(23) $$\Delta P^{g}=\int \int {\dot{V}}^{g}(t)dt=\int \int (C_{b}^{g}f_{ib}^{b}(t)-(2\omega_{ie}^{g}(t)+\omega_{eg}^{g}(t))\times V^{g}(t)+G^{g})dtdt,$$

where $f_{ib}^{b}$ is the specific force in the body frame *b*, $\omega_{ie}^{g}$ is the angular rate of the earth frame e to the inertial frame i, $\omega_{eg}^{g}$ is the angular rate of the local geographical frame g to the Earth frame e, $V^{g}$ is the velocity and $G^{g}$ is the gravity vector. The superscript indicates the frame that these vectors are projected into. $C_{b}^{g}$ is the direction cosine matrix of transformation from frame b to frame g.

The values of $\omega_{ie}^{g}$ and $G^{g}$ relate to the longitude and latitude, while the value of $\omega_{eg}^{g}$ is a function of the longitude, latitude and $V^{g}$. Practically, the changes of the longitude and latitude will not influence the values of $\omega_{ie}^{g}$, $G^{g}$ and $\omega_{eg}^{g}$ during the GPS outages as they would not last too long. Thus, the effect of the changes of the position is relatively quite small comparing to other factors. $C_{b}^{g}$ can be denoted as:

(24) $$C_{b}^{g}=\left[\begin{array}{ccc} \cos R\cos H+\sin R\sin H\sin P & \sin H\cos P & \sin R\cos H-consR\sin H\sin P\\ \sin R\cos H\sin P-\cos R\sin H & \cos H\cos P & -\sin R\sin H-\cos R\cos H\sin P\\ -\sin R\cos P & \sin P & \cos R\cos P \end{array}\right],$$

where *P*, *R,* and *H* are pitch, roll, and yaw respectively. For land vehicles, the body frame is always in the local level, where *P* and *R* are 0° $C_{b}^{g}$ is determined by *H*. Thus, the specific force, velocity, and yaw information are employed as the inputs of AI module, and the increments of GPS position are predicted to provide the pseudo-GPS position data.

**Figure 1:**
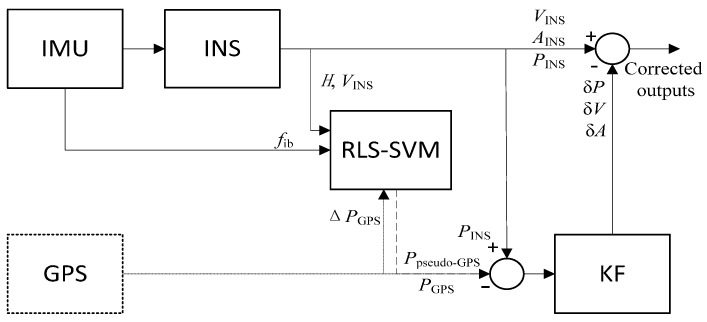
RLS-SVM aided INS/GPS integrated navigation system.

Figure 1 illustrates the configuration of the RLS-SVM aided INS/GPS integrated navigation system. The dotted lines shows the integration process when GPS is available, while the dash lines indicate the information fusion procedure during the GPS outages. The other parts work all of the time. *P*, *V*, and *A* are position, velocity, and attitude, respectively, and δ indicates the error. When GPS data is available, the whole system is in a loosely-coupled mode and the RLS-SVM module is in the training mode. The outputs of INS and GPS are integrated by KF, where the attitude, velocity, and position errors are estimated as a correction to the hybrid system. Meanwhile, the specific force, velocity, and yaw information is sent to the RLS-SVM module as the inputs, while the increments of the GPS position are given as the expected outputs. Since the expected outputs only contain the GPS information, the RLS-SVM can easily figure out the outliers. When GPS is unavailable, the specific force, velocity, and yaw information is still provided for the RLS-SVM module and a prediction of the increments of GPS position can be obtained. After the integral, the pseudo-GPS position information could be achieved, which is then used as the input of the KF to form the observation vector with the INS position. The hybrid system will continuously give the integrated information during the GPS outages.

The RLS-SVM algorithm is used to deal with the situation when GPS information is still available but introduces some errors. Rather than share the same weight in the traditional LS-SVM, the proposed algorithm allocates various weights for different data, which makes the system immune to the outliers. In the training mode, the inputs and the expected outputs of RLS-SVM form the training dataset ${\left\{x_{k},y_{k}\right\}}_{k=1}^{N}$ to train the RLS-SVM network, where N denotes the total number of seconds of the training period, $x_{k}$ and $y_{k}$ are the *k*th input and output vectors. When GPS is unavailable, the whole system switches to the prediction mode, where $x_{k}$ is still sent to the well-trained RLS-SVM network. The predicted results can then be achieved by Equation (19). The detailed operation is illustrated in next section.

In this work, the historical information is explored to better represent the vehicle dynamics. Not only the specific force, velocity, and yaw information in the current moment, but also those in the last few seconds, is considered to form the input vector together with the current data. The number of the past steps that should be involved is investigated in the next section.

## 5. Test Results

Field test data were collected on a land vehicle platform to evaluate the proposed algorithm. A prototype of INS which consisted of three fiber optic gyroscopes and three quartz accelerometers was utilized, where the gyro bias was 0.01°/h and the accelerometer accuracy was 50 μg. The FlexPark6 GPS receiver was from NovAtel (Calgary, AB, Canada). Meanwhile, PHINS from IXBLUE (Saint-Germain en Laye, France) was utilized as a reference system to provide the accurate navigation information.

**Figure 2:**
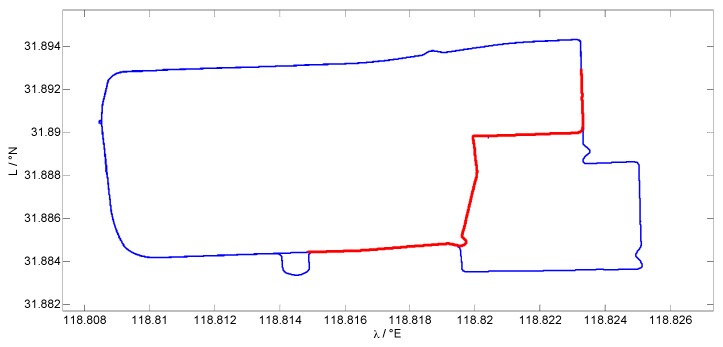
Vehicle trajectory.

Figure 2 shows the coordinates of the vehicle trajectory, which was conducted at the Jiulonghu campus of Southeast University in Nanjing. The red line indicates the assumed GPS outage, which lasts for 300 s. After the 900 s alignment period, the whole system is performed under the loosely-coupled mode. The GPS data are integrated with INS information to give a consistent, relatively high accuracy, navigation result, during which the AI module using the RLS-SVM algorithm is trained to map the relationship between the vehicle dynamics and the position increments. The vehicle dynamics are described by the velocity, yaw, and specific force data in the current moment and last second, which are regarded as the input vector of the AI module. The position increments calculated from the GPS position data are treated as the expected output vector of the AI module. Given both the input and output vectors, which are denoted as x and y, respectively, apply the RLS-SVM algorithm to train the AI network. First, let the weight matrix S equal the identity matrix I and calculate the parameter α and *b* according to Equation (16), then reusing the input vector x and the parameter α and *b*, calculate the regression result f(x) according to Equation (19). The difference between the regression result f(x) and the output vector y is regarded as the residual vector, which contains residuals in each second. According to the statistics theory, if a certain residual is larger than three times that of the standard deviation, it is regarded as an outlier and the corresponding sample data in the training set should be eliminated. After recognizing the obvious outliers in the training set and deleting them, recalculate the parameter α and *b*, after which the weight matrix S is updated by Equation (21) to further reduce the remaining outlier effect by decreasing the weight of those samples with large residuals. Once the updated weight matrix S is obtained, the final α and *b* can be achieved by (16). From 3200 s to 3500 s, the GPS signal is supposed to be unavailable and the AI module switches to the prediction mode. The same kind of information is inputted into the AI module, including the velocity, yaw, and specific force data in the current moment and last second, to form the new input vector x. Then, the well-trained AI network will calculate the corresponding output by Equation (19), using α, *b*, and the old input vectors set $\left\{x_{i}\right\}$. After the integral, the predicted position is achieved to be regarded as the pseudo-GPS data, fusing with the INS by the KF. The hybrid system will provide the integrated information during the GPS outage continuously.

**Figure 3:**
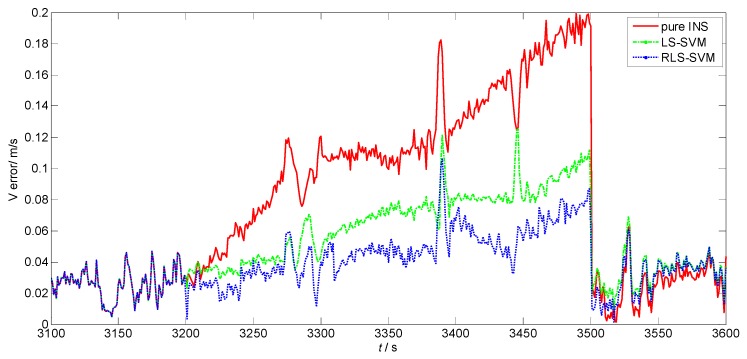
Velocity errors among different algorithms.

**Figure 4:**
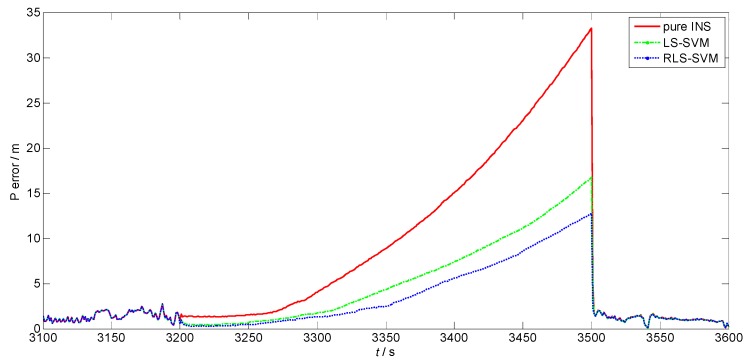
Position errors among different algorithms.

Both the LS-SVM algorithm and the pure INS are compared with the RLS-SVM method to evaluate its performance. As the altitude damping method is employed by the system, only horizontal errors are involved to make a judgment. Figure 3 and Figure 4 present velocity errors and position errors among different algorithms from 3100 s to 3600 s, respectively. Before 3200 s, the GPS signal is still available and the whole system is in the loosely-coupled mode, where the three methods show the same navigation result. From 3200 s to 3500 s, the GPS is unavailable. The performance of the three methods varies. The red lines in both figures indicate the navigation results of the pure INS mode, while the green dashed lines and blue dotted lines denote the performance of the LS-SVM and RLS-SVM algorithms, respectively. It can be seen that the proposed RLS-SVM method outperforms the LS-SVM method, which is much better than the pure INS mode. The velocity and position accuracy of all the three navigation solutions deteriorates with time during the GPS outage. At 3500 s, the horizontal velocity errors of the pure INS, LS-SVM and RLS-SVM are 0.198 m/s, 0.1115 m/s, and 0.0869 m/s, while the position errors are 33.26 m, 16.72 m, and 12.72 m, respectively. We can see that during the GPS outage, the proposed algorithm achieves good performance when the vehicle operates in this irregular trajectory. The LS-SVM algorithm, which suffers from the GPS outliers, cannot reach the best model to predict the position increments. At the end of the GPS outage, the velocity and position errors of the RLS-SVM algorithm are only about 40% of the pure INS method and 75% of the LS-SVM method.

When the GPS signal is recovered at 3500 s, the whole system switches to the loosely-coupled mode again. The position error and velocity error quickly converges and the navigation results of the three methods are approaching the same along with time. As the position difference of the INS and the GPS are directly the observation vector of the KF, the position errors of the three methods has little difference after 3500 s, while the velocity errors differ slightly, but converge towards one another.

During the training period, where the first 2300 s data is trained and investigated, the 2300 s data is separated into two data sets. The training set includes the data from 900 s to 3000 s, which is used to train the LS-SVM and RLS-SVM models. The validation set involves the data from 3001s to 3200 s, which is utilized to validate the effectiveness of the trained models. Thus, the effectiveness of the prediction of the position increments of the two models can be compared on the validation set, which is shown below.

**Figure 5:**
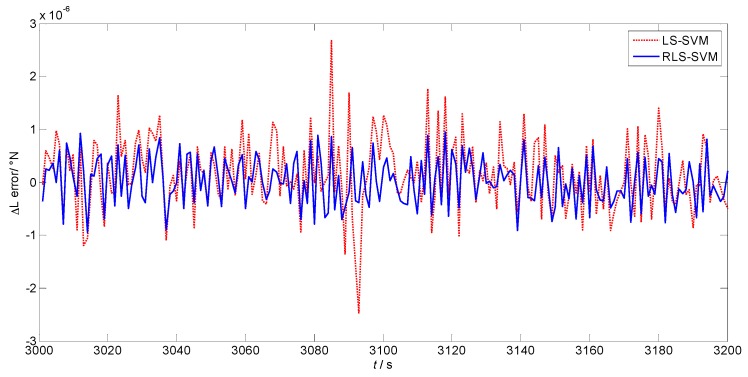
Prediction error of the position increment in latitude.

**Figure 6:**
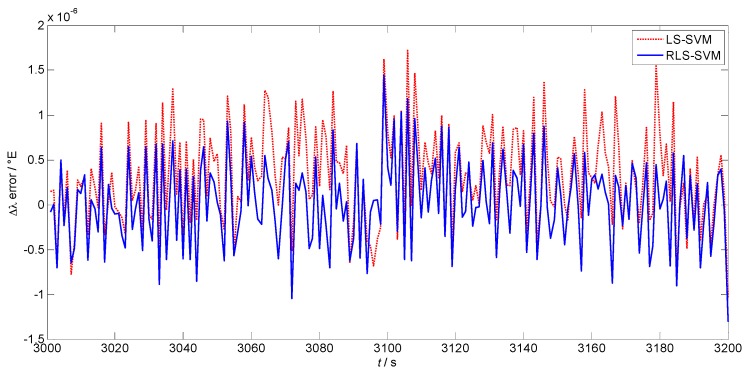
Prediction error of the position increment in longitude.

Figure 5 and Figure 6 are prediction errors of the position increment of the two algorithms in latitude and longitude, respectively. The red dotted lines are the results of LS-SVM, while the blue lines show the performance of the proposed algorithm. The mean values of the prediction error using LS-SVM and RLS-SVM are $1.548\times 10^{-70}$ and $2.225\times 10^{-80}$ in Figure 5, while the standard deviations are $6.825\times 10^{-70}$ and $4.538\times 10^{-70}$, respectively. The mean values of the prediction error in longitude are $3.271\times 10^{-70}$ and $3.236\times 10^{-80}$, while the standard deviations are $5.356\times 10^{-70}$ and $4.798\times 10^{-70}$, respectively. It can be concluded that the proposed algorithm makes more stable and more accurate position predictions than the LS-SVM algorithm.

**Figure 7:**
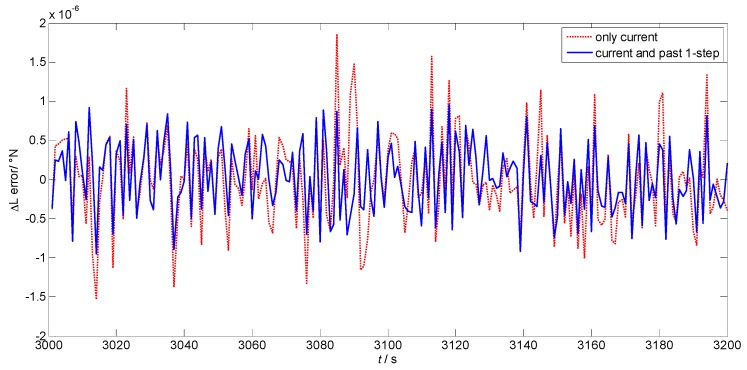
Prediction error of the position increment in latitude using different steps.

**Figure 8:**
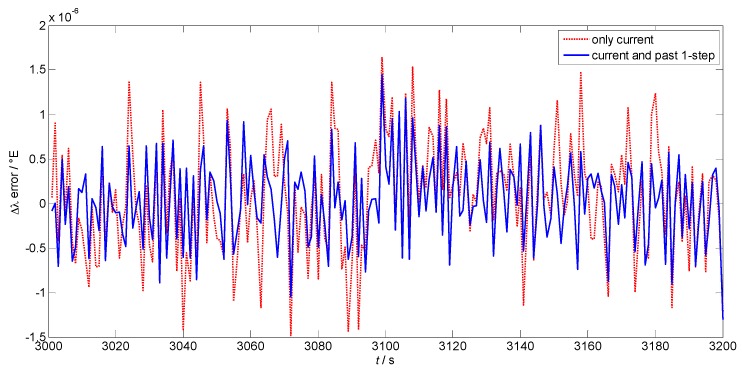
Prediction error of the position increment in longitude using different steps.

In previous studies, the historical data was also employed to better describe the dynamic situation and make a more accurate prediction [18,19,20]. However, the steps of the past data should be carefully selected, which varies in different models and applications. In this study, the number of the steps of the past data are considered from 0 to 2, where 0 means only the current data is employed. Figure 7 and Figure 8 shows the prediction errors of the position increments in latitude and longitude using different steps of the past data. The red dotted lines are the results when only the current data is used to train the RLS-SVM module. The blue lines are the results when both the current and the past one-step information is utilized. In addition, the similar analysis is conducted involving two steps of the past data. The mean values and the standard deviations of the prediction errors using different steps are listed in Table 1.

We can see that when both the current and past one-step data are used, the prediction accuracy is higher than that when only current information is employed. It can be concluded that the current data cannot represent the vehicle dynamic alone and the past data must be involved to better illustrate the demanding information. Meanwhile, the results of involving past one-step and past two-step data are similar, which means that the past one-step data has already given good performance. Thus, considering the computation complexity, the current and past one-step data are selected as the inputs of the proposed algorithm.

Additionally, to reduce the computational complexity, the inputs of the AI module are simplified. As illustrated in Section 4, only yaw information among the three attitudes is employed. Meanwhile, because the land vehicle was operated on a smooth trajectory without hilly roads, only the horizontal velocities and specific forces are considered. The similar simulation is also done to make a comparison of the simplified solution and the full inputs solution. The mean values of the prediction errors of simplified solution and full inputs solution in latitude are $2.225\times 10^{-80}$ and $2.205\times 10^{-80}$, while the standard deviations are $4.538\times 10^{-70}$ and $4.853\times 10^{-70}$. The mean values of the prediction errors in longitude are $3.236\times 10^{-80}$ and $3.233\times 10^{-80}$, while the standard deviations are $4.798\times 10^{-70}$ and $4.802\times 10^{-70}$, respectively. Thus, the simplified solution is adopted in this study as it can achieve almost the same prediction accuracy as the full input solution.

**Figure 9:**
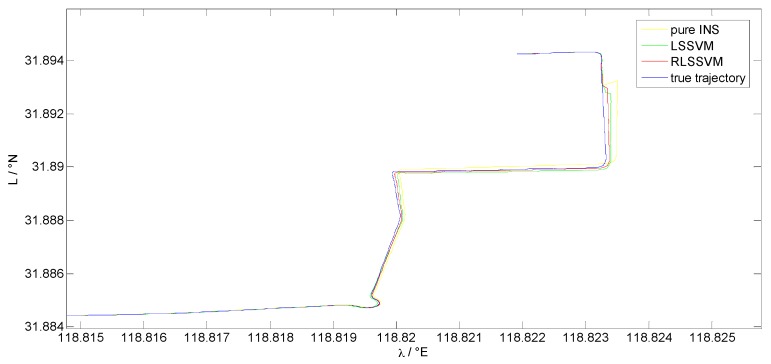
Positioning results of different algorithms.

Figure 9 shows the positioning results of the pure INS method, LS-SVM method, and the proposed algorithm. The true trajectory is also plotted to make a comparison. It can be seen that the proposed algorithm could provide a relatively higher accuracy solution to bridge the GPS outage.

## 6. Conclusions

The AI-aided INS solution to bridge the GPS outage has been a newly raised hotspot to relieve the navigation problems when GPS is unavailable. When GPS is available, the AI module is trained, which will be employed to make predictions of the demanding information during GPS outages. During the training process, the GPS data collected for training is always assumed with high accuracy. However, in real applications, there are outliers among the GPS information which cannot be easily recognized. To relieve the negative effect of the outliers on the AI module, a robust learning algorithm should be explored and its corresponding AI model should be constructed.

In this study, an improved AI aided solution is proposed to solve the positioning problems during the GPS outages. When the GPS signal is available, the whole system is under the loosely-coupled mode. The information of the yaw, specific force, velocity, and position increments are collected to try to investigate the inner relationship between them. When GPS data is unavailable, the well-trained AI module will continuously provide prediction of the position increments to form pseudo-GPS position data to provide corrections to the standalone INS. Furthermore, a RLS-SVM regression algorithm is developed to deal with the outliers in the training dataset. Based on the theory of statistics, the training data are allocated different weights according to their residuals, which makes the outliers much less important compared to the other data. Field test data was collected to evaluate the performance of the proposed method. It can be seen that, during the 300 s GPS outage, the proposed RLS-SVM-aided navigation solution outperforms the LS-SVM solution, and is much better than the pure INS mode. Additionally, the proposed algorithm can make better prediction of the position increments than the LS-SVM method, which shows its talent in reducing the negative effect of the GPS outliers. Finally, the inputs of the RLS-SVM are simplified. The current and past one-step data are selected to represent the vehicle dynamics well.

## Figures and Tables

**Table 1:** Prediction errors of different steps.

Inputs of the AI Module	Mean Value in Latitude (°)	Standard Deviation in Latitude (°)	Mean Value in Longitude (°)	Standard Deviation in Longitude (°)
Current information only	$-1.392\times 10^{-8}$	$5.676\times 10^{-7}$	$7.611\times 10^{-8}$	$6.503\times 10^{-7}$
Current and past one-step	$2.225\times 10^{-8}$	$4.538\times 10^{-7}$	$3.236\times 10^{-8}$	$4.798\times 10^{-7}$
Current and past two-steps	$1.611\times 10^{-8}$	$4.53\times 10^{-7}$	$3.866\times 10^{-8}$	$4.768\times 10^{-7}$
